# Right-Sided Extravalvular Damage: An Overlooked Driver of Risk Model Failure in Low-Flow, Low-Gradient Aortic Stenosis

**DOI:** 10.1016/j.jscai.2025.104116

**Published:** 2025-12-30

**Authors:** Yash Prakash, Akarsh Sharma, Lakshay Chopra, Mihir Prakash, Eileen Galvani, Oludamilola Akinmolayemi, Carlo Mannina, Ranbir Singh, Jonathan L. Halperin, Samin K. Sharma, Annapoorna S. Kini, Deepak L. Bhatt, Stamatios Lerakis

**Affiliations:** a Samuel Bronfman Department of Medicine, Icahn School of Medicine at Mount Sinai, New York, New York; b Department of Cardiology, Mount Sinai Morningside and Mount Sinai West, New York, New York; c College of Arts and Sciences, Case Western Reserve University, Cleveland, Ohio; d Mount Sinai Fuster Heart Hospital, Icahn School of Medicine at Mount Sinai, New York, New York

**Keywords:** low-flow low-gradient aortic stenosis, patient selection, right-sided extravalvular damage, risk prediction, transcatheter aortic valve replacement

## Abstract

**Background:**

The Society of Thoracic Surgeons Predicted Risk of Mortality (STS-PROM) score is guideline-endorsed for estimating procedural risk in aortic stenosis (AS) but tends to overestimate long-term mortality after transcatheter aortic valve replacement (TAVR). This study aimed to evaluate whether markers of right-sided extravalvular damage improve STS-PROM’s performance in predicting 1-year mortality post-TAVR for patients with low-flow, low-gradient AS.

**Methods:**

Four hundred ten patients with valve area ≤1.0 cm^2^, stroke volume index ≤35 mL/m^2^, and mean gradient <40 mm Hg who underwent TAVR were retrospectively stratified into low (<4%), moderate (4%-8%), and high (>8%) STS-PROM risk groups. Performance of an enhanced logistic regression model incorporating both STS-PROM and right-sided extravalvular damage (defined as moderate+ pulmonary hypertension, moderate+ tricuspid regurgitation, or right ventricular systolic dysfunction) was assessed using receiver operating characteristic curves, net reclassification index (NRI), and integrated discrimination index (IDI).

**Results:**

Right-sided extravalvular damage was independently associated with increased 1-year mortality (OR, 2.45; 95% CI, 1.34-4.46; *P* < .01). Model fit improved with its addition (Δχ^2^ = 8.68, *P* < .01), and discrimination increased (IDI = 0.02, *P* = .01). NRI analysis showed improved survivor classification (non-event NRI = 0.10, *P* < .01). The largest gain was in moderate-risk patients (area under the curve, 0.46-0.67; *P* = .06).

**Conclusions:**

Markers of right-sided extravalvular damage conferred meaningful prognostic value in low-flow, low-gradient AS by correcting the STS-PROM score’s tendency to overestimate post-TAVR mortality. The added value of these markers was most pronounced in moderate-risk patients, for whom existing tools appear least reliable. These markers may improve patient selection for TAVR and merit evaluation in other severe AS phenotypes.

## Introduction

The use of transcatheter aortic valve replacement (TAVR) has expanded dramatically over the past decade, transforming the management of severe aortic stenosis (AS) across nearly all surgical risk profiles.^1^ As TAVR is applied to broader patient populations, there remains a critical need for accurate risk stratification tools to guide long-term prognostication and clinical decision-making.

Current American College of Cardiology and American Heart Association guidelines recommend the Society of Thoracic Surgeons Predicted Risk of Mortality (STS-PROM) score to estimate procedural risk.^2^ Although the STS-PROM model incorporates a wide range of clinical variables—including patient demographic characteristics, comorbidities, cardiac function, coronary anatomy, and operative details—it was only validated to predict the 30-day mortality risk after surgical aortic valve replacement.^3^ Subsequent studies have demonstrated that STS-PROM tends to overestimate mortality risk after TAVR and performs suboptimally in predicting longer-term outcomes, thereby limiting its utility for guiding patient selection and risk stratification in contemporary TAVR populations.^4^, ^5^, ^6^

Emerging evidence highlights the prognostic importance of extravalvular cardiac damage. In 2017, Généreux et al^7^ proposed a damage staging system, demonstrating that patients with right-sided involvement—defined by pulmonary hypertension, tricuspid regurgitation, or right ventricular (RV) dysfunction—had significantly higher mortality following TAVR in the PARTNER 2 trials. These findings were recently validated by Nakase et al^8^ in 2024 using the SwissTAVI registry. Despite its broad scope, the STS-PROM model does not account for right-sided extravalvular damage, raising the possibility that integrating such markers could improve long-term risk prediction and, in turn, enhance overall clinical decision-making for TAVR.

To investigate whether markers of right-sided extravalvular damage improve risk prediction, we focused on patients with low-flow, low-gradient (LFLG) AS, a high-risk subgroup characterized by precarious hemodynamics and increased mortality after TAVR.^9^^,^^10^ In LFLG AS, conventional markers such as mean gradient and indexed stroke volume may be discordant not only with true disease severity but also with overall clinical risk.^11^ Given the vulnerability of this population, patients with LFLG AS offer a relevant and high-yield setting in which to assess the additive prognostic value of right-sided damage for long-term outcomes following TAVR.

## Materials and methods

### Study design and patient population

This retrospective cohort study included consecutive adult patients (age ≥18 years) evaluated for TAVR at a large quaternary-care hospital in New York City between January 2019 and December 2022. All patients underwent standardized 2-dimensional and Doppler transthoracic echocardiography, interpreted by structural heart disease echocardiographers in accordance with American Society of Echocardiography guidelines.^12^^,^^13^

Patients were included if they had severe LFLG AS, defined as an aortic valve area ≤1.0 cm^2^, a stroke volume index ≤35 mL/m^2^, and a mean transvalvular gradient <40 mm Hg, and subsequently underwent TAVR during the study period. To ensure complete and unbiased end point ascertainment, patients who were lost to follow-up before 12 months post-TAVR without confirmed death (verified through the Limited Access Death Master File) were excluded from all analyses, including model-building, discrimination assessments, and Kaplan-Meier survival analyses.

Transcatheter aortic valve replacement candidacy, procedural approach, and device selection were determined by a multidisciplinary heart team based on individualized assessments, including cardiovascular comorbidities (eg, coronary artery disease, valvular disease, arrhythmias), functional status (by Kansas City Cardiomyopathy Questionnaire-12 scores and New York Heart Association class), frailty indicators (eg, chronic kidney disease), and perioperative mortality risk estimated by the STS-PROM score. Devices used included the SAPIEN 3 (Edwards Lifesciences); CoreValve, Evolut R, and Evolut PRO (Medtronic); and Acurate neo and Acurate neo2 (Boston Scientific).

Baseline demographic characteristic, clinical, and echocardiographic data were extracted retrospectively from the electronic medical record. The study protocol was approved by the Institutional Review Board at the Icahn School of Medicine at Mount Sinai, with a waiver of informed consent due to the retrospective design.

### Primary end point

The primary end point was all-cause mortality following TAVR. Mortality status postprocedure was determined through electronic medical record review and cross-referencing with the Limited Access Death Master File. One-year all-cause mortality was used as the outcome for logistic regression modeling, receiver operating characteristic curve analysis, and net reclassification index (NRI) and integrated discrimination index (IDI) calculations. For Kaplan-Meier survival analyses, overall survival was assessed from the date of TAVR to death or last known clinical follow-up within the cohort of patients with verified 1-year outcome data.

### Statistical analysis

Right-sided extravalvular damage was assessed using a custom variable adapted from Généreux et al^7^ and Nakase et al,^8^ defined by the presence of moderate or greater pulmonary hypertension, moderate or greater tricuspid regurgitation, and/or RV systolic dysfunction. Moderate pulmonary hypertension was defined by a pulmonary artery systolic pressure >40 mm Hg, as assessed by right heart catheterization (preferred) or by echocardiographic estimation when invasive data were unavailable. RV systolic dysfunction was documented as the presence of at least 1 of the following: tricuspid annular plane systolic excursion <1.7 cm, tissue Doppler S′ velocity <9.5 cm/s, or RV fractional area change <35%. If more than 2 RV parameters were available and discordant, dysfunction was adjudicated by prioritizing tricuspid annular plane systolic excursion, followed by S′ velocity, and then fractional area change.

Baseline demographic characteristic, clinical, and echocardiographic characteristics were summarized as medians with interquartile ranges for continuous variables and as counts with percentages for categorical variables. Normality of continuous variables was assessed using the Shapiro-Wilk test. Group comparisons were performed using 1-way analysis of variance for normally distributed continuous variables, Kruskal-Wallis tests for non-normally distributed continuous variables, and Pearson’s χ^2^ tests for categorical variables.

Kaplan-Meier survival analyses were performed to compare overall survival between patients with high versus low right-sided damage within each STS-PROM risk stratum, defined as low (<4%), moderate (4%-8%), and high (≥8%) predicted 30-day mortality risk. Patients alive at the last known follow-up were censored at the time of last contact. To account for characteristics known to affect outcomes after TAVR, propensity score matching was performed using a logistic regression model incorporating the following covariates: age, sex, coronary artery disease requiring coronary artery bypass grafting or percutaneous coronary intervention, chronic kidney disease, prior cerebrovascular accident, moderate-or-worse mitral regurgitation, atrial fibrillation, moderate-or-worse paravalvular leak, 30-day pacemaker implantation, severe prosthesis-patient mismatch, and LFLG AS subtype (classical vs paradoxical).^14^, ^15^, ^16^, ^17^, ^18^, ^19^ After matching, univariate Cox proportional hazards models were used to estimate hazard ratios (HR) for 1-year mortality between right-sided damage groups within each STS-PROM stratum.

Three logistic regression models were developed to predict 1-year mortality: (1) a baseline model including STS-PROM score alone; (2) an enhanced risk model incorporating STS-PROM score and our composite variable representing right-sided extravalvular damage; and (3) an enhanced risk model including STS-PROM score along with each component of right-sided damage (moderate or greater pulmonary hypertension, moderate or greater tricuspid regurgitation, and RV dysfunction).

Performance of the baseline and enhanced risk model was assessed with 4 complementary analytic approaches. To limit optimistic bias from assessing enhanced model performance with the same cohort used to develop the model, nonparametric bootstrap resampling with 1000 replications was used for performance metrics when possible. First, model fit was assessed using likelihood ratio tests and pseudo-R^2^ values. Associations between individual predictors and the outcome were quantified using odds ratios with 95% CI. Second, discrimination was evaluated using receiver operating characteristic curves, with area under the curve (AUC) values calculated both overall and within each STS-PROM 30-day mortality risk stratum. Third, calibration was evaluated using 3 complementary methods to assess different aspects of model accuracy: calibration-in-the-large (comparing overall observed versus predicted mortality rates), calibration slope (assessing systematic overconfidence or underconfidence across risk levels), and Hosmer-Lemeshow goodness-of-fit tests stratified by STS-PROM risk groups to identify stratum-specific calibration performance. Calibration plots were constructed using risk deciles for the overall cohort and quintiles or quartiles for individual strata based on sample size considerations. Lastly, reclassification performance was quantified using NRI analysis. Patients were classified into predefined risk categories based on STS-PROM alone and then reclassified using the enhanced model. NRI components were calculated separately for events (deaths) and nonevents (survivors). To complement NRI, the IDI was calculated to assess changes in overall model discrimination.

All statistical analyses were performed using Stata version 18.5 (StataCorp). A 2-sided *P* value <.05 was considered statistically significant, and *P* values ≥.05 and <.10 were interpreted as suggestive trends.

## Results

### Baseline characteristics

A total of 410 patients with severe LFLG AS who underwent TAVR were included in the analysis. Baseline demographic characteristic, clinical, and echocardiographic characteristics stratified by right-sided extravalvular damage are presented in Table 1.

**Table 1:** Demographic, clinical, and echocardiographic baseline characteristics stratified by STS-PROM tertiles.

Characteristics	Low-risk (STS-PROM <4%) (n = 227)			Moderate-risk (STS-PROM 4%-8%) (n = 125)			High-risk (STS-PROM ≥8%) (n = 58)		
	Right-sided damage		*P* value	Right-sided damage		*P* value	Right-sided damage		*P* value
	Low (n = 162)	High (n = 65)		Low (n = 66)	High (n = 59)		Low (n = 20)	High (n = 38)	
Age, y	78 (73-83)	79 (73-82)	.79	84 (78-88)	84 (75-89)	.78	89 (83-92)	88 (82-93)	.77
BMI, kg/m^2^	28 (25-32)	28 (24-31)	.98	27 (24-31)	26 (23-28)	.22	27 (24-28)	25 (23-28)	.47
Male sex	104 (64.2%)	50 (76.9%)	.06	33 (50.0%)	36 (61.0%)	.22	11 (55.0%)	24 (63.2%)	.55
CAD	83 (51.2%)	37 (56.9%)	.44	48 (72.7%)	36 (61.0%)	.16	16 (80.0%)	31 (81.6%)	>.99
Prior CABG	17 (10.5%)	7 (10.8%)	.95	17 (25.8%)	16 (27.1%)	.86	4 (20.0%)	18 (47.4%))	.04^a^
Prior PCI	57 (35.2%)	27 (41.5%)	.37	39 (59.1%)	23 (39.0%)	.03^a^	14 (70.0%)	25 (65.8%)	.75
Prior CVA	30 (18.5%)	12 (18.5%)	.99	20 (30.3%)	10 (17.0%)	.08	2 (10.0%)	10 (26.3%)	.19
PAD	16 (9.9%)	4 (6.2%)	.37	15 (22.7%)	10 (17.0%)	.42	5 (25.0%)	9 (23.7%)	>.99
HTN	150 (92.6%)	58 (89.2%)	.41	59 (89.4%)	59 (100%)	.01^a^	19 (95.0%)	35 (92.1%)	>.99
HLD	153 (94.4%)	58 (89.2%)	.25	62 (93.9%)	54 (91.5%)	.73	18 (90.0%)	35 (92.1%)	>.99
DM	72 (44.4%)	25 (38.5%)	.41	30 (45.5%)	22 (37.3%)	.36	8 (40.0%)	12 (31.6%)	.52
CKD	30 (18.5%)	23 (35.4%)	.01^a^	38 (57.6%)	25 (42.4%)	.09	11 (55.0%)	27 (71.1%)	.22
Liver disease	6 (3.7%)	2 (3.1%)	>.99	2 (3.0%)	1 (1.7%)	>.99	1 (5.0%)	1 (2.6%)	>.99
AFib	39 (24.1%)	30 (46.2%)	<.01^a^	16 (24.2%)	37 (62.7%)	<.01^a^	5 (25.0%)	26 (68.4%)	<.01^a^
BNP, pg/mL	107 (46-222)	303 (150-736)	<.01^a^	157 (82-369)	410 (237-893)	<.01^a^	278 (148-397)	1050 (341-1920)	<.01^a^
Cr, mg/dL	0.9 (0.7-1.1)	1.0 (0.9-1.4)	<.01^a^	1.2 (0.9-1.7)	1.1 (0.9-1.3)	.31	1.2 (0.9-1.6)	1.4 (1.0-2.3)	.15
Antiplatelet	121 (74.7%)	43 (66.2%)	.19	51 (77.3%)	36 (61.0%)	.05^a^	15 (75.0%)	26 (68.4%)	.60
Anticoagulation	37 (22.8%)	33 (50.8%)	<.01^a^	14 (21.2%)	37 (62.7%)	<.01^a^	4 (20.0%)	20 (52.6%)	.02^a^
ACEi/ARB	75 (46.3%)	26 (40.0%)	.39	33 (50.0%)	18 (30.5%)	.03	10 (50.0%)	9 (23.7%)	.04^a^
ARNI	5 (3.1%)	10 (15.4%)	<.01^a^	4 (6.1%)	4 (6.8%)	>.99	0 (0.0%)	3 (7.9%)	.54
BB	88 (54.3%)	48 (73.9%)	.01^a^	42 (63.6%)	43 (72.9%)	.27	13 (65.0%)	29 (76.3%)	.36
CCB	46 (28.4%)	14 (21.5%)	.29	20 (30.3%)	15 (25.4%)	.54	9 (45.0%)	6 (15.8%)	.02^a^
Loop diuretic	28 (17.3%)	33 (50.8%)	<.01^a^	24 (36.4%)	33 (55.9%)	.03^a^	8 (40.0%)	23 (60.5%)	.13
MRA	8 (4.9%)	7 (10.8%)	.14	3 (4.6%)	9 (15.3%)	.04	1 (5.0%)	2 (5.3%)	>.99
SGLT2i	10 (6.2%)	7 (10.8%)	.26	5 (7.6%)	5 (8.5%)	>.99	0 (0.0%)	4 (10.5%)	.29
CRT	5 (3.1%)	2 (3.1%)	>.99	3 (4.6%)	5 (8.5%)	.47	0 (0%)	3 (7.9%)	.54
ICD	5 (3.1%)	2 (3.1%)	>.99	4 (6.1%)	10 (17.0%)	.05^a^	0 (0%)	6 (15.8%)	.08
NYHA class 3+	118 (72.8%)	47 (72.3%)	.94	58 (87.9%)	54 (91.5%)	.51	19 (95.0%)	35 (92.1%)	>.99
KCCQ-12	32 (26-37)	29 (25-34)	.04^a^	30 (24-32)	26 (19-32)	.02^a^	29 (25-33)	25 (20-31)	.09
STS-PROM, %	2.4 (1.6-3.1)	2.7 (2.1-3.2)	.01^a^	4.9 (4.4-6.1)	5.3 (4.5-6.2)	.63	9.3 (8.8-11.9)	11.4 (9.5-13.5)	.08
LVEF, %	62 (55-65)	45 (35-58)	<.01^a^	60 (47-65)	50 (38-61)	<.01^a^	65 (54-66)	37 (30-55)	<.01^a^
AVA, cm^2^	0.72 (0.62-0.84)	0.71 (0.59-0.85)	.81	0.71 (0.62-0.82)	0.73 (0.64-0.84)	.49	0.69 (0.64-0.78)	0.65 (0.60-0.74)	.31
iAVA, cm^2^/m^2^	0.38 (0.32-0.42)	0.35 (0.31-0.40)	.29	0.40 (0.33-0.44)	0.40 (0.36-0.45)	.32	0.39 (0.34-0.47)	0.39 (0.34-0.43)	.46
Aortic peak velocity, m/s	3.5 (3.2-3.8)	3.3 (2.9-3.6)	<.01^a^	3.3 (2.9-3.7)	3.2 (2.7-3.5)	.19	3.1 (3.0-3.7)	3.1 (2.8-3.5)	.39
Aortic MG, mm Hg	29 (22-34)	27 (20-30)	.09	25 (18-32)	22 (17-27)	.13	22 (19-30)	22 (18-28)	.45
SVi, mL/m^2^	29.5 (26.6-33.2)	27.2 (22.0-30.0)	<.01^a^	30.1 (26.6-32.9)	28.4 (22.3-32.2)	.03^a^	29.3 (25.7-33.6)	27.5 (25.1-29.6)	.06
Indexed LA diameter, mm/m^2^	20.6 (17.8-23.0)	22.3 (20.5-25.7)	.01^a^	22.7 (19.5-27.2)	26.4 (24.6-30.0)	.03^a^	19.1 (18.4-20.1)	25.6 (23.9-31.4)	<.01^a^
Estimated PASP, mm Hg	28 (22-32)	44 (36-57)	<.01^a^	28 (25-34)	46 (40-56)	<.01^a^	24 (22-37)	56 (49-63)	<.01^a^
>Moderate MR	8 (4.9%)	14 (21.5%)	<.01^a^	12 (18.2%)	19 (32.2%)	.07	3 (15.0%)	14 (36.8%)	.08
>Moderate TR	0 (0%)	10 (15.4%)	<.01^a^	0 (0.0%)	27 (45.8%)	<.01^a^	0 (0%)	21 (55.3%)	<.01^a^
>Moderate AR	6 (3.7%)	5 (7.7%)	.30	3 (4.6%)	2 (3.4%)	>.99	0 (0.0%)	2 (5.3%)	.54
RV dysfunction^b^	0 (0.0%)	46 (70.8%)	<.01^a^	0 (0.0%)	34 (57.6%)	<.01^a^	0 (0.0%)	27 (71.1%)	<.01^a^
Verified true-severe^c^	82 (50.6%)	33 (50.8%)	.98	25 (37.9%)	26 (44.1%)	.48	7 (35.0%)	20 (52.6%)	.20
Verified pseudo-severe^c^	76 (46.9%)	30 (46.1%)	.92	39 (59.1%)	31 (52.5%)	.58	12 (60.0%)	14 (36.8%)	.09
Classical LFLG AS	25 (15.4%)	39 (60.0%)	<.01^a^	18 (27.3%)	28 (47.5%)	.02^a^	4 (20.0%)	26 (68.4%)	<.01^a^
Paradoxical LFLG AS	137 (84.6%)	26 (40.0%)	<.01^a^	48 (72.7%)	31 (52.5%)	.02^a^	16 (80.0%)	12 (31.6%)	<.01^a^

Values are median (IQR) or n (%).

ACEi, angiotensin-converting enzyme inhibitor; AFib, atrial fibrillation; AR, aortic regurgitation; ARB, angiotensin receptor blocker; ARNI, angiotensin receptor–neprilysin inhibitor; AS, aortic stenosis; AVA, aortic valve area; BB, beta-blocker; BMI, body mass index; BNP, B-type natriuretic peptide; CABG, coronary artery bypass grafting; CAD, coronary artery disease; CCB, calcium channel blocker; CKD, chronic kidney disease; Cr, creatinine; CRT, cardiac resynchronization therapy; CVA, cerebrovascular accident; DM, diabetes mellitus; HLD, hyperlipidemia; HTN, hypertension; iAVA, indexed aortic valve area; ICD, implantable cardioverter defibrillator; KCCQ-12, Kansas City Cardiomyopathy Questionnaire-12; LFLG, low-flow, low-gradient; LVEF, left ventricular ejection fraction; MG, mean gradient; MR, mitral regurgitation; MRA, mineralocorticoid receptor antagonist; NYHA, New York Heart Association; PAD, peripheral artery disease; PCI, percutaneous coronary intervention; RV, right ventricular; SGLT2i, sodium-glucose cotransporter-2 inhibitors; STS-PROM, Society of Thoracic Surgeons Predicted Risk of Mortality; SVi, indexed stroke volume; TR, tricuspid regurgitation.

a Statistically significant difference (*P* < .05).

b RV dysfunction was documented as the presence of at least 1 of the following parameters: tricuspid annular plane systolic excursion <1.7 cm, doppler S′ <9.5 cm/s, or fractional area change <35%. If more than 2 parameters were available and discrepant, RV dysfunction was defined by prioritizing in the order of tricuspid annular plane systolic excursion, S′, then fractional area change.

c Classification of true-severe versus pseudo-severe AS was determined using dobutamine stress echocardiography and/or aortic valve calcium scoring by computed tomography, when available.

Several notable trends were observed across STS-PROM risk strata. Patients with high right-sided extravalvular damage had a significantly higher prevalence of atrial fibrillation and larger indexed left atrial diameters compared with those with low right-sided damage (*P* < .01 for both across all strata). B-type natriuretic peptide levels were also elevated in patients with high right-sided damage (*P* < .01 for all), and anticoagulation use was more common in this group (*P* < .01 in low- and moderate-risk, *P* = .02 in high-risk).

Echocardiographically, patients with high right-sided damage had lower indexed stroke volumes (*P* < .01 in low-risk, *P* = .03 in moderate-risk, and *P* = .06 in high-risk) and lower left ventricular (LV) ejection fraction across all strata (*P* < .01 for all). These findings were accompanied by a higher prevalence of classical LFLG AS among patients with high right-sided damage, whereas paradoxical LFLG AS was more common in those with low right-sided damage (*P* < .01 for both in low- and high-risk strata and *P* = .02 for both in moderate-risk groups). There were no significant differences in indexed aortic valve area, peak aortic valve velocities, or mean transvalvular gradients between groups.

### Kaplan-Meier survival analyses

**Figure 1:**
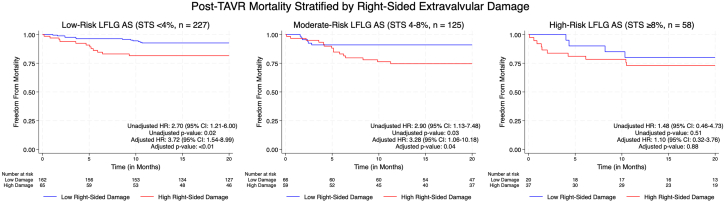
**Prognostic value of right-sided extravalvular damage in low-flow, low-gradient (LFLG) aortic stenosis (AS) patients undergoing transcatheter aortic valve replacement (TAVR).** Kaplan-Meier survival curves showing freedom from 1-year all-cause mortality in patients with LFLG AS undergoing TAVR, stratified by the Society of Thoracic Surgeons Predicted Risk of Mortality (STS-PROM) risk tertiles: low-risk (<4%, left), moderate-risk (4%-8%, center), and high-risk (≥8%, right). Within each group, patients were further stratified by right-sided extravalvular damage burden (low vs high). Adjusted hazard ratios (HR) and *P* values were derived from Cox regression models following propensity score matching using covariates including age, sex, coronary artery disease requiring coronary artery bypass grafting (CABG) or percutaneous coronary intervention, chronic kidney disease, prior cerebrovascular accident, atrial fibrillation, moderate/severe mitral regurgitation, severe paravalvular leak, 30-day permanent pacemaker implantation, severe prosthesis-patient mismatch, and LFLG AS subtype. Right-sided damage was independently associated with higher mortality in the low- and moderate-risk strata.

Kaplan-Meier survival analyses stratified by right-sided extravalvular damage stage demonstrated significant differences in 1-year mortality within both the low-risk and moderate-risk STS-PROM groups (Figure 1). The median follow-up duration was 2.5 years.

Propensity score matching successfully matched 333 of 410 (81%) patients, including 127 patients with high right-sided damage and 206 patients with low right-sided damage, achieving excellent covariate balance with all standardized differences <10%. In the low-risk group, patients with high right-sided damage had significantly higher 1-year mortality (adjusted HR, 3.72; 95% CI, 1.54-8.99; *P* < .01), as did those in the moderate-risk group (adjusted HR, 3.28; 95% CI, 1.06-10.18; *P* = .04). In contrast, no significant difference in mortality was observed within the high-risk group (adjusted HR, 1.10; 95% CI, 0.32-3.76; *P* = .88).

### Enhanced model development and assessment

In the baseline logistic regression model including STS-PROM score alone, the pseudo-R^2^ was 0.03 (likelihood ratio χ^2^ = 10.98, *P* < .01) (Table 2). In the enhanced model incorporating a composite variable representing right-sided extravalvular damage, model performance improved (pseudo-R^2^ = 0.06; χ^2^ = 19.66, *P* < .01), with a statistically significant improvement in model fit compared to the baseline model (Δχ^2^ = 8.68, *P* < .01) (Central Illustration). Within this enhanced model, right-sided extravalvular damage was independently associated with greater than 2-fold increased odds of 1-year mortality (OR, 2.45; 95% CI, 1.34-4.46, *P* < .01). In a separate model including each component variable—moderate or greater pulmonary hypertension, moderate or greater tricuspid regurgitation, and RV dysfunction—only RV dysfunction remained independently predictive of 1-year mortality (OR, 2.20; 95% CI, 1.16-4.18, *P* = .02).

**Table 2:** Logistic regression models predicting 1-year mortality.

Model	Predictors	OR (95% CI)	*P* value	LR χ^2^ (df)	Pseudo-R2
Original	STS-PROM score	1.12 (1.05-1.20)	<.01^a^	10.98^b^ (1)	0.03
Enhanced with composite variable	STS-PROM score Right-sided extravalvular damage	1.09 (1.01-1.17) 2.45 (1.34-4.46)	.02^a^ <.01^a^	19.66^b^ (2)	0.06
Enhanced with component variables	STS-PROM score Moderate+ pHTN^c^ Moderate+ tricuspid regurgitation Right ventricular dysfunction^d^	1.10 (1.02-1.18) 1.70 (0.83-3.48) 0.67 (0.27-1.64) 2.20 (1.16-4.18)	.01^a^ .15 .38 .02^a^	20.83 (4)	0.06

LR, likelihood ratio; OR, odds ratio; pHTN, pulmonary hypertension; STS-PROM, Society of Thoracic Surgeons Predicted Risk of Mortality.

a Statistically significant difference (*P* < .05).

b Δχ^2^ (improvement from original to composite-enhanced model): 8.68 (df = 1), *P* < .01.

c Moderate pHTN was defined by pulmonary artery systolic pressure >40 mm Hg, as assessed by right heart catheterization (preferred) or by echocardiographic estimation when invasive data were unavailable.

d Right ventricular dysfunction was documented as the presence of at least 1 of the following parameters: tricuspid annular plane systolic excursion <1.7 cm, doppler S′ <9.5 cm/s, or fractional area change <35%. If more than 2 parameters were available and discrepant, right ventricular dysfunction was defined by prioritizing in the order of tricuspid annular plane systolic excursion, S′, then fractional area change.

**Central Illustration:**
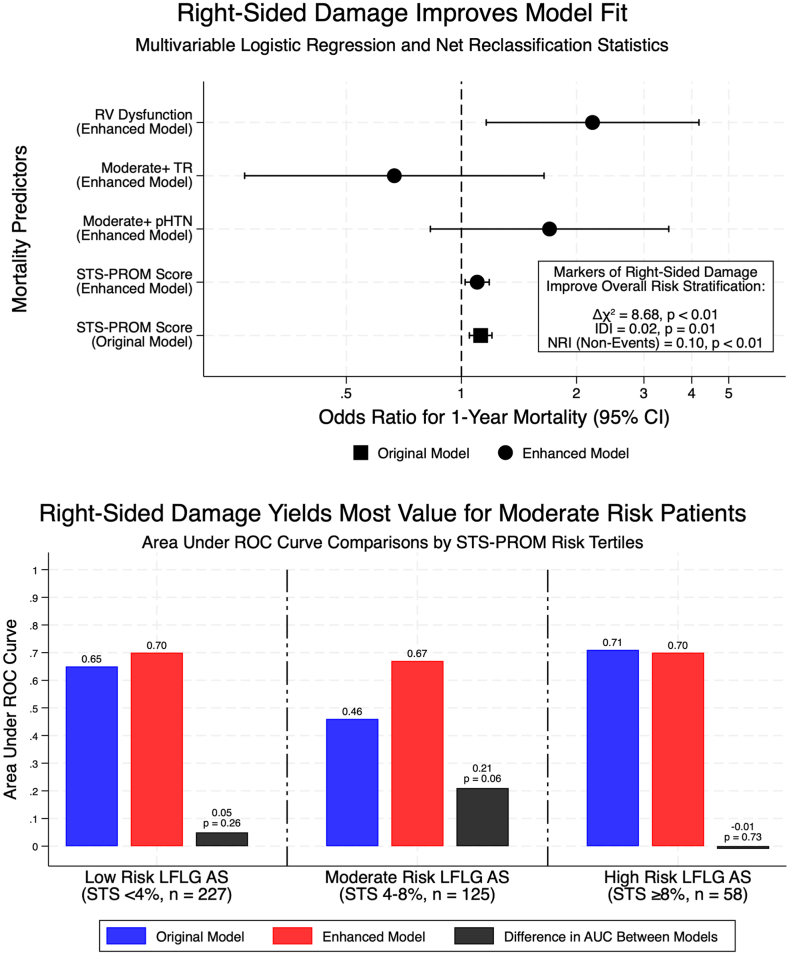
Top panel: Forest plot displaying odds ratios (OR) for 1-year all-cause mortality from logistic regression models. The baseline model included the Society of Thoracic Surgeons Predicted Risk of Mortality (STS-PROM) score alone. Two enhanced models were constructed: one incorporating a composite variable for right-sided extravalvular damage (defined as the presence of moderate or greater pulmonary hypertension [pHTN], moderate or greater tricuspid regurgitation [TR], and/or right ventricular [RV] systolic dysfunction), and a second model by entering these components individually. Of the individual predictors, only RV dysfunction was independently associated with mortality (OR, 2.20; 95% CI 1.16-4.18; *P* = .02). Incorporating the composite right-sided damage variable significantly improved model fit (Δχ^2^ = 8.68, *P* < .01), nonevent reclassification (non-event net reclassification index [NRI] = 0.10, *P* < .01), and overall discrimination (integrated discrimination index [IDI] = 0.02, *P* = .01). Error bars represent 95% CI. Bottom panel: Bar graph comparing area under the receiver operating characteristic curve (AUC) for 1-year mortality prediction using the baseline STS-PROM model versus the enhanced model incorporating right-sided extravalvular damage. Results are stratified by STS-PROM risk tertiles: low (<4%), moderate (4%-8%), and high (≥8%). Discrimination improved most in the moderate-risk group (AUC, 0.46-0.67; Δ = 0.21, *P* = .06), with minimal changes in low-risk (Δ = 0.05, *P* = .26) and high-risk groups (Δ = –0.01, *P* = .73). AUC and *P* values reflect bootstrapped ROC analysis. AS, aortic stenosis; LFLG, low-flow, low-gradient.

**Figure 2:**
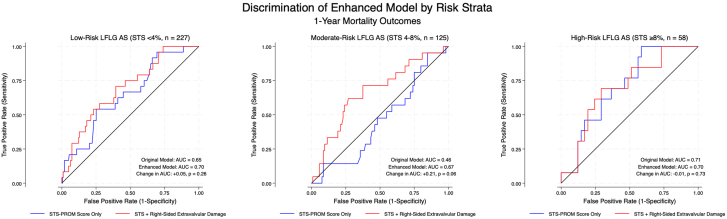
**Discriminative capacity of the enhanced model in low-flow, low-gradient (LFLG) aortic stenosis (AS) patients undergoing transcatheter aortic valve replacement.** Receiver operating characteristic curves comparing model discrimination for 1-year mortality across Society of Thoracic Surgeons Predicted Risk of Mortality (STS-PROM) risk strata. The baseline model included STS-PROM score alone; the enhanced model added a composite variable for right-sided extravalvular damage. In the moderate-risk group, the enhanced model showed a trend toward improved discrimination (area under the curve [AUC] 0.67 vs 0.46; Δ = 0.21, *P* = .06), with smaller or no improvements in the low-risk (Δ = +0.05, *P* = .26) and high-risk groups (Δ = –0.01, *P* = .73). AUC comparisons used DeLong tests on bootstrapped receiver operating characteristic curves.

Discrimination analysis revealed that the baseline model with STS-PROM score alone exhibited poor discrimination for 1-year mortality, particularly within the moderate-risk group (Figure 2). In the moderate-risk group, the baseline AUC for STS-PROM score alone was 0.46 (95% CI, 0.33-0.59). After incorporating right-sided extravalvular damage into our enhanced model, the AUC increased to 0.67 (95% CI, 0.53-0.80), reflecting a suggestive trend toward improved discrimination (*P* = .06). In the overall cohort, the AUC improved from 0.63 (95% CI, 0.56-0.71) with STS-PROM alone to 0.69 (95% CI, 0.61-0.75) after incorporating right-sided damage.

**Figure 3:**
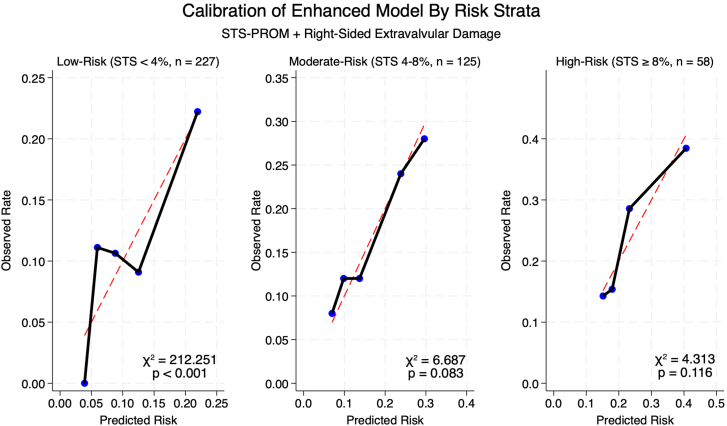
**Calibration of enhanced model in low-flow, low-gradient aortic stenosis patients undergoing transcatheter aortic valve replacement.** Model calibration was assessed across Society of Thoracic Surgeons Predicted Risk of Mortality (STS-PROM) risk strata using calibration plots and Hosmer-Lemeshow goodness-of-fit tests. Points closer to the diagonal line indicate better calibration within each stratum. Although the enhanced model overall demonstrated excellent calibration-in-the-large (observed-expected = 0.003) and optimal calibration slope (1.000, *P* > .99), subanalysis by risk strata revealed heterogeneous calibration performance. The enhanced model maintained good calibration in both moderate-risk (STS 4%-8%; χ^2^ = 6.687, *P* = .083) and high-risk patients (STS ≥8%; χ^2^ = 4.313, *P* = .116), but demonstrated poor calibration in low-risk patients (STS <4%; χ^2^ = 212.251, *P* < .001).

Calibration testing revealed heterogeneous performance of the enhanced model (Figure 3). The overall model demonstrated excellent calibration-in-the-large (observed-expected = 0.003) and optimal calibration slope (1.000, *P* > .99). When stratified by STS-PROM risk strata, the enhanced model maintained good calibration in both moderate-risk (χ^2^ = 6.687, *P* = .083) and high-risk patients (χ^2^ = 4.313, *P* = .116) but demonstrated poor calibration in the low-risk patients (χ^2^ = 212.251, *P* < .001).

Reclassification analysis by NRI and IDI analyses revealed a statistically significant positive non-event NRI (0.10; 95% CI, 0.04-0.16; *P* < .01), indicating improved correct classification of survivors (Table 3). The neutral event NRI (0.00; 95% CI, –0.14 to 0.14; *P* > .99) suggested no significant impact on the classification of deaths. The total NRI was positive (0.10; 95% CI, –0.05 to 0.25) but did not reach statistical significance (*P* = .20). The IDI was 0.02 (95% CI, 0.00-0.03; *P* = .01), reflecting overall improved discrimination with the enhanced model.

**Table 3:** Net reclassification analysis of enhanced model (STS-PROM + right-sided cardiac damage).

Metric	Value (95% CI)	*P* value
Event NRI	0.00 (–0.14 to 0.14)	>.99
Non-event NRI	0.10 (0.04 to 0.16)	<.01^a^
Total NRI	0.10 (–0.05 to 0.25)	.20
IDI	0.02 (0.00 to 0.03)	.01^a^

IDI, integrated discrimination index; NRI, net reclassification index.

a Statistically significant difference (*P* < .05).

## Discussion

In this retrospective cohort study of patients with LFLG AS undergoing TAVR, we found that right-sided extravalvular damage provided incremental prognostic information beyond the STS-PROM score. Patients with evidence of right-sided damage who were initially classified as those with low- or moderate-risk by STS-PROM had survival curves more closely resembling those of patients in the high-risk tertile than those of their counterparts without right-sided damage within the same STS-PROM tertile (Figure 1). Incorporating markers of right-sided damage improved the prediction of 1-year all-cause mortality, as evidenced by enhanced logistic regression model performance (likelihood ratio χ^2^ improvement = 8.68, *P* < .01; pseudo-R^2^ increase from 0.03 to 0.06). IDI analysis confirmed improved overall model discrimination (IDI = 0.02, *P* = .01). Notably, the greatest improvement in AUC was observed in the moderate-risk STS-PROM tertile, where the AUC increased from 0.46 to 0.67 (*P* = .06). Although this specific statistic indicated a suggestive trend toward improved discrimination within this specific subgroup that did not reach the traditional threshold for statistical significance, likely due to limited sample size, the overall findings consistently support the value of incorporating right-sided damage to refine risk prediction in LFLG AS (Central Illustration).

A notable finding was the significant positive non-event NRI (0.10, *P* < .01) when the enhanced model incorporated right-sided extravalvular damage. This improvement in correctly classifying survivors suggests that right-sided damage helped correct the known tendency of the STS-PROM scores to overestimate long-term mortality risk described in prior TAVR populations.^4^, ^5^, ^6^ This is likely because current surgical risk models integrate a broad set of demographic, comorbidity, and procedural risk factors but do not fully capture the extent of chronic cardiac remodeling, and thus less accurately predict outcomes after TAVR.^3^ Incorporating clinical assessment of right-sided cardiac damage alongside existing risk models may, therefore, more accurately stratify TAVR candidates by risk.

Among the individual components of the enhanced model, RV systolic dysfunction was most strongly associated with 1-year mortality, underscoring its central prognostic importance. In contrast, other markers of right-sided extravalvular damage, such as tricuspid regurgitation and pulmonary hypertension, were not independently associated with mortality in our cohort. These findings suggest that although such features may reflect progressive remodeling, it is overt RV dysfunction that ultimately drives worse post-TAVR outcomes.

In our cohort, patients with high right-sided extravalvular damage demonstrated a greater burden of LV systolic dysfunction overall, including lower left ventricular ejection fraction, reduced stroke volume index, and higher prevalence of classical LFLG AS. These patients were also more likely to exhibit atrial fibrillation, elevated B-type natriuretic peptide levels, and larger indexed left atrial diameters—markers of left atrial and upstream remodeling. Together, these findings align with the framework proposed by Généreux et al,^7^ which describes sequential, time-dependent cardiac remodeling in the setting of valvular heart disease, beginning with the left heart and ultimately extending to the right heart. The strong association between RV dysfunction and mortality highlights the need for timely identification and intervention before biventricular remodeling occurs.

The greatest improvement in model discrimination after incorporating right-sided extravalvular damage was observed within the moderate-risk STS-PROM group. This finding may carry particular clinical significance, as decision-making in moderate-risk patients can be more nuanced and uncertain. Although low- and high-risk TAVR candidates are more easily triaged based on clinical gestalt and guideline-driven recommendations, patients classified as those with moderate-risk likely represent a more heterogeneous group in whom outcome prediction is less reliable. Our findings raise the possibility that incorporating right-sided damage assessment could be most valuable for this subgroup, where traditional models and clinical intuition offer limited guidance.

It is important to note that the STS-PROM score was designed to estimate surgical mortality risk and cannot fully substitute for comprehensive TAVR risk assessment. Some patients may be considered high-risk for TAVR despite low STS-PROM scores due to clinical considerations such as vascular access or unfavorable aortic root and valve morphology. Conversely, patients considered high-risk for surgical AVR by STS-PROM may remain suitable TAVR candidates. Thus, although the addition of right-sided extravalvular damage markers enhances prognostic accuracy of the STS-PROM model, individualized evaluation by a multidisciplinary heart team remains essential to guide therapeutic decision-making and optimize outcomes for TAVR.

Several limitations warrant consideration. First, this was a small, retrospective, single-center study limited to patients with LFLG AS who underwent TAVR, introducing inherent selection bias and limiting generalizability to the broader population of patients with LFLG AS. Prospective multicenter research is needed to validate these findings in more representative cohorts. Second, our study may have limited statistical power to detect effects in subanalyses by risk tertiles. Larger studies are needed to definitively assess the utility of right-sided extravalvular damage across all risk strata and rule out type II error. Third, because our analysis focused exclusively on LFLG AS, it remains unknown whether the prognostic utility of right-sided extravalvular damage extends to patients with high-gradient or normal-flow AS. This is an important question for future research. Fourth, although the STS-PROM model predicts a range of perioperative outcomes, including stroke, renal failure, reoperation, prolonged ventilation, and hospital length of stay, our study focused exclusively on 1-year all-cause mortality.^3^ We did not assess these additional outcomes, and whether right-sided damage also influences them remains an important area for future investigation. Fourth, although the 1-year follow-up provides important prognostic insights, this timeframe may be insufficient to fully characterize the impact of right-sided damage on long-term remodeling, symptom burden, and clinical outcomes. Lastly, we did not evaluate quality-of-life metrics, functional capacity, or heart failure hospitalizations, which are increasingly recognized as critical end points in this high-risk population. Future research incorporating comprehensive clinical and patient-reported outcomes, as well as longer-term follow-up, is needed to validate and extend our findings.

## Conclusion

Right-sided extravalvular damage confers incremental prognostic value beyond the STS-PROM score in patients with LFLG AS undergoing TAVR. Its inclusion improves model performance, particularly in moderate-risk patients, for whom existing tools and clinical judgment may be less reliable. Importantly, right-sided damage ameliorates the known tendency of the STS-PROM score to overestimate long-term mortality, enhancing discrimination and improving risk assessment classification. Although these findings are specific to LFLG AS, they underscore the broader potential of integrating right-sided damage markers into TAVR risk assessment. Future studies should evaluate whether these insights extend to other severe AS phenotypes and support more accurate, individualized patient selection.
